# Efficacy of the Radiotherapy on Darier's Disease: An Indirect Evidence

**DOI:** 10.1155/2013/907802

**Published:** 2013-09-03

**Authors:** Ala Podgornii, Patrizia Ciammella, Dafne Ramundo, Cinzia Iotti

**Affiliations:** Radiation Therapy Unit, Department of Oncology and Advanced Technology, Azienda Ospedaliera ASMN, Istituto di Ricovero e Cura a Carattere Scientifico, Viale Risorgimento 80, 42121 Reggio Emilia, Italy

## Abstract

Darier's disease (DD) is an autosomal dominant dermatosis characterized by hyperkeratotic papules that are mainly located in the seborrheic areas and pushups, handheld wells, and nails. The disease often appears at a young age, typically by the third decade, with no sex predilection. There is currently no standard therapy and there are usually topical palliative therapies. We present the case of an affected 42-year-old woman treated with radiation therapy for early breast cancer. Before the radiotherapy, the patient showed hyperkeratotic, brownish papules extending in a linear pattern for the neck to the abdomen, especially on both breasts and inframammary area. During the radiation, she developed grade 1 to 2 dermatitis in the irradiated area. At a followup of 6 months, the patient has no skin lesions in the irradiated zone. This report suggests that the radiotherapy is not contraindicated and may indeed be effective in local control of skin lesions in DD.

## 1. Introduction

Darier's disease (DD), also known as Darier-White disease [1], Dyskeratosis follicularis [2], and keratosis follicularis [3], is an autosomal dominant dermatosis characterized by hyperkeratotic papules that are particularly dense in the seborrheic areas and flexures, palmar pits, and nail dystrophy.

Darier's disease affects both men and women and is not contagious. The disease often appears at a young age, typically by the third decade, with no sex predilection. It is considered a rare disease because its prevalence in the world is estimated to be between 1 : 30.000 and 1 : 100.000.

It most commonly affects the chest, neck, back, ears, and groin but may involve other body areas. The rash associated with Darier's disease often has a distinct odour. Palms and soles may become thickened, and intraoral papules can be found. Finger nails become fragile, and this helps in diagnosis of the disease. The rash can be aggravated by heat, humidity, and exposure to sunlight. In some cases, sunlight makes it better, especially in the forehead.

About etiopathogenesis, it was reported that mutations in the gene AT-P2A2, located on chromosome 12q23-24.1, cause DD [4]. It seems likely that the mutation interferes with the normal internal calcium signalling that regulates processes such as cell proliferation, differentiation, and adhesion between keratinocytes [5]. This defect in intracellular calcium regulation justifies histological features, including dyskeratosis, suprabasal acantholysis, and papillomatosis.

DD is difficult to treat. A variety of palliative medical therapies exist as topical emollients containing urea or lactic acid [6], antibiotics, topical steroids, and topical retinoids [7, 8].

Occasional reports showed an initial and transient response to radiotherapy [9–11].

## 2. Case Report

We report the case of a 42-year-old woman who presented in our clinic due to recent diagnosis of early left breast cancer. The computer tomography performed revealed no other organs compromised or distant metastatic extension. The patient did not have relevant medical history of interest, except for a Darier's disease known for many years.

The patient underwent a lumpectomy with axillary lymph node dissection. The pathological diagnosis was moderately differentiated invasive ductal carcinoma (IDC) of the right breast of 2.3 cm in size with metastasis detected in 1/18 lymph nodes removed (pT_2_ pN_1 _M_0_). Immunohistochemical study of the tumor cells showed positive staining for both estrogen receptors (ER) and progesterone receptors (PR) in 95% and 95%, respectively, and showed positive membrane staining of HER-2 marker. With this diagnosis, the patient received postoperative adjuvant chemotherapy consisting of 6 cycles of FEC followed by maintaining treatment with trastuzumab for 1 year and adjuvant hormone therapy with tamoxifen 20 mg daily during the following 5 years. Then, the patient was referred to our radiation oncology department where she received adjuvant radiotherapy (50 Gy) on left-breast residual tissue.

She was primarily diagnosed of DD in 1991 with pruritic eruption on the trunk. There was no family history of a similar skin summer. A biopsy specimen revealed acantholytic dyskeratotic cells in the upper epidermis, suprabasal clefts with focal hyperkeratosis, and parakeratosis which were consistent with the clinical diagnosis of Darier's disease. Various therapeutic options had been applied: lotions containing cortisone, estrogens, or testosterone and several homeopathic options. Due to progression disease, the patient was treated with oral retinoids (acitretin: initial 10 mg) plus cyclosporine, obtaining an initial good response with disappearance of the skin lesions. After nine months using systemic retinoid and cyclosporine, the papules have reappeared in almost the whole body. This systemic therapy is still ongoing, but the patient occasionally required the topical corticosteroids during acute flares.

**Figure 1:**
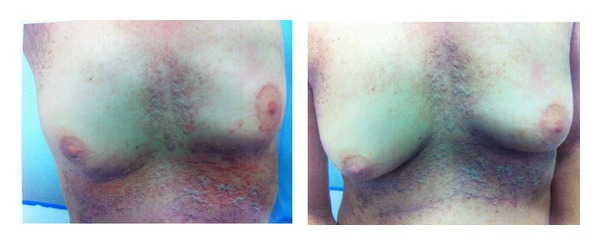
Skin features at diagnosis.

**Figure 2:**
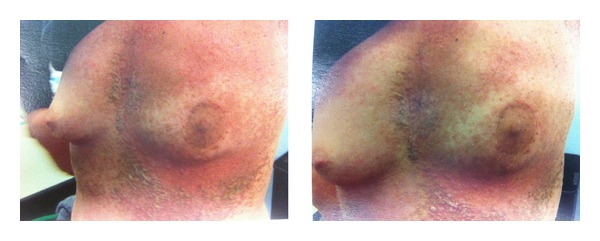
Skin features during radiation therapy.

Before the radiotherapy, the patient showed hyperkeratotic, brownish papules extending in a linear pattern from the neck to the abdomen, especially on both breasts and inframammary area (Figure 1). During the radiation, she developed grade 1 to 2 dermatitis in the irradiated area (Figure 2).

**Figure 3:**
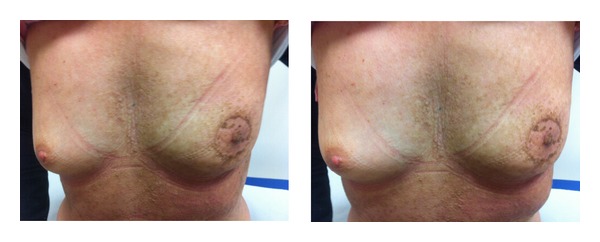
Skin features after 1 month.

**Figure 4:**
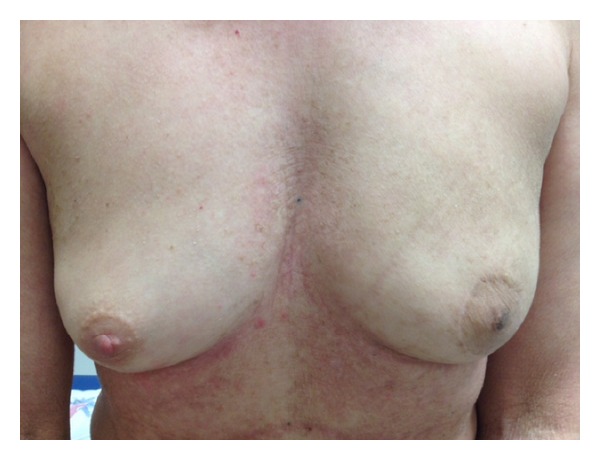
Skin features after 6 months.

One month after the end of radiotherapy, the patient showed a good remission of the skin lesions in the irradiated zone (Figure 3). At a followup of 6 months, the patient has no skin lesions in the irradiated zone (Figure 4).

## 3. Discussion

Darier's disease is a dominantly inherited skin condition, characterized by hyperkeratotic papules coalescing to warty patches on symmetrical areas of the face, trunk, and flexures of the extremities. It has long been known that the DD is an autosomal dominant disorder; the responsible mutations have been identified on chromosome 12q23-24.1. The gene encodes a calcium-ATPase type 2 in the sarco-/endoplasmic reticulum (SERCA2) which belongs to the large family of P-type cation pumps. This pump couples ATP hydrolysis to the transport of cations across membranes and thus plays a significant role in intracellular calcium signalling.

Darier's disease is difficult to treat and has no known cure. A variety of palliative medical therapies exist for DD as topical creams, antibiotics, topical steroids, and topical retinoids.

Other treatments have included psoralen and ultraviolet A, CO_2_ vaporization, cryosurgery, and photodynamic therapy. This last treatment, as highlighted in a recent study of six patients, can be viewed as a potential adjunctive modality for Darier's disease but should not be considered as a substitute for retinoids in patients who require systemic treatment [12].

Currently, the treatment is strictly limited to the relief of symptoms. In severe cases, oral retinoids (acitretin: initial 10–20 mg/Tag and isotretinoin: 0.5–1 mg/kg/day) lead to a response in 90% of cases but often the disease remission is transient.

The role of radiotherapy in this setting of patients is unknown. Sporadic studies have reported a beneficial outcome in terms of disappearance of skin lesions. Beier and Kaufmann [9] reported the outcome of six patients affected by Darier's disease and Hailey-Hailey disease treated with radiotherapy. At median followup of 9 months, the treated areas did not show skin lesions. Two other reports [10, 11] revealed an initial transient beneficial response to radiation treatment.

Mac Manus et al. [13] reported a case of patient with Darier's disease treated with radiation therapy for bronchial cancer. This patient during and immediately after radiotherapy showed a severe exacerbation of Darier's skin lesions in the irradiated area but subsequently had a complete and permanent disappearance.

Two recent experiences described the efficacy of electron beam radiotherapy in Darier's disease [14, 15]. The first [14] used a dose of 2 Gy daily with energy of 7.5 MeV generated by a linear accelerator. At the followup, the irradiated areas remained without recurrence, whereas new lesions in untreated body sites developed. The second study [15] reported a case of severe DD that was poorly responsive to known therapeutic modalities. This patient was treated with a total dose of 20 Gy in 10 fractions using 6 MeV electrons. The patient initially developed a severe local dermatitis with desquamation. In a few weeks, the skin lesions regressed, and the irradiated areas remained free from skin recurrence.

Our patient, treated for breast cancer, had benefited from radiotherapy also for what concerns Darier's disease. Our single experience, together with piecemeal reports, suggests that radiotherapy is not contraindicated and may indeed be effective in local control of skin lesions in poorly responsive DD. Then, local radiotherapy can be used for localized areas of severe, recalcitrant symptomatic disease. The doses and techniques need to be investigated in prospective studies.
